# Dynamic epigenetic states of maize centromeres

**DOI:** 10.3389/fpls.2015.00904

**Published:** 2015-10-26

**Authors:** Yalin Liu, Handong Su, Jing Zhang, Yang Liu, Fangpu Han, James A. Birchler

**Affiliations:** ^1^State Key Laboratory of Plant Cell and Chromosome Engineering, Institute of Genetics and Developmental Biology, Chinese Academy of SciencesBeijing, China; ^2^University of Chinese Academy of SciencesBeijing, China; ^3^Division of Biological Sciences, University of Missouri at Columbia, ColumbiaMO, USA

**Keywords:** centromere inactivation, centromere pairing, non-disjunction, *de novo* centromere, neocentromere, epigenetics, maize

## Abstract

The centromere is a specialized chromosomal region identified as the major constriction, upon which the kinetochore complex is formed, ensuring accurate chromosome orientation and segregation during cell division. The rapid evolution of centromere DNA sequence and the conserved centromere function are two contradictory aspects of centromere biology. Indeed, the sole presence of genetic sequence is not sufficient for centromere formation. Various dicentric chromosomes with one inactive centromere have been recognized. It has also been found that *de novo* centromere formation is common on fragments in which centromeric DNA sequences are lost. Epigenetic factors play important roles in centromeric chromatin assembly and maintenance. Non-disjunction of the supernumerary B chromosome centromere is independent of centromere function, but centromere pairing during early prophase of meiosis I requires an active centromere. This review discusses recent studies in maize about genetic and epigenetic elements regulating formation and maintenance of centromere chromatin, as well as centromere behavior in meiosis.

## Introduction

The centromeres are the control centers of chromosomes and are essential for correct orientation and segregation in cell division. The kinetochore complex formation requires a functional centromere, so that the spindle can attach to the centromere regions for accurate orientation and segregation of chromosomes (Cleveland et al., 2003; Allshire and Karpen, 2008).

Centromeres can be divided into three types according to their structural organization: the point centromere with a 125 bp single nucleosome in *Saccharomyces cerevisiae*, the regional centromeres with several kilobases to megabases of repeat sequences in most organisms, and the holocentromeres that are spread throughout the chromosome as in *Caenorhabditis elegans* (Allshire and Karpen, 2008) and the genus Luzula (Heckmann et al., 2013). In plants, most centromeres are regional centromeres; the sizes are larger and the repeat sequences are more complicated than other species (Zhang and Dawe, 2012; Feng et al., 2015).

In maize (*Zea mays*), there are mainly two kinds of centromeric repeat sequences, namely a 156 bp tandem repeat CentC and centromeric retrotransposon of maize (CRM; Ananiev et al., 1998; Zhong et al., 2002). During evolution, maize centromere sizes and the arrangement of centromere sequences have experienced insertion, deletion, duplication, and other changes (Wang and Bennetzen, 2012). The B chromosome centromere in maize has an additional specific entromere repeat sequence called the B-repeat (Alfenito and Birchler, 1993) that surrounds and is interspersed with the CentC and CRM arrays (Jin et al., 2005; Lamb et al., 2005). The pericentromere sequence, Cent4, on chromosome 4 in maize has some sequence similarity to the B-repeat sequence (Page et al., 2001). In the second pollen mitosis, the functional B centromere undergoes non-disjunction, leading to both sister-chromatids segregating to the same pole (Carlson, 2007). Several elements encoded by various regions on the B chromosome act on the B centromere to perform non-disjunction (Lin, 1978). Centromere misdivision of the B centromere of the TB-9Sb translocation line in maize, which is a reciprocal translocation between the B chromosome and the short arm of chromosome 9, can produce derivatives with changed centromere sizes and DNA sequences (Kaszás and Birchler, 1998).

The functional centromere associates with a specific histone H3 variant, which is a heritable epigenetic marker of centromere identity. It was first identified in human and called centromere protein (CENP)-A in 1985 (Earnshaw and Rothfield, 1985). In plants, the centromeric H3 variants (CENH3) have also been identified in maize and other plants including *Arabidopsis* (Talbert et al., 2002; Zhong et al., 2002). There are two copies of CENH3 in barley, wheat, *Pisum*, and *Lathyrus*, which may have functions in polyploidy formation and chromosome evolution (Ishii et al., 2015; Neumann et al., 2015; Yuan et al., 2015). Another histone modification marker for functional centromeres in maize is phosphorylation at Thr133 in histone H2A (Dong and Han, 2012). The centromeric sequences CentC and CRM in maize interact with CENH3-nucleosomes (Zhong et al., 2002).

Centromere function is not determined by DNA sequence. As described below, several examples of inactive centromeres have been documented in maize as well as several examples of *de novo* centromere formation over unique sequences. Taken together, this evidence suggests an epigenetic basis of centromere specification in maize. In this review, we will discuss current studies and potential mechanisms in centromere formation, centromere activity maintenance and special centromere behavior in maize.

## Centromere Inactivation

Dicentric chromosomes can be produced through chromosome translocation. Chromosomes with two active centromeres are usually unstable during the cell cycle. Therefore stable dicentric chromosomes have only one functional centromere with the other one inactive.

**FIGURE 1:**
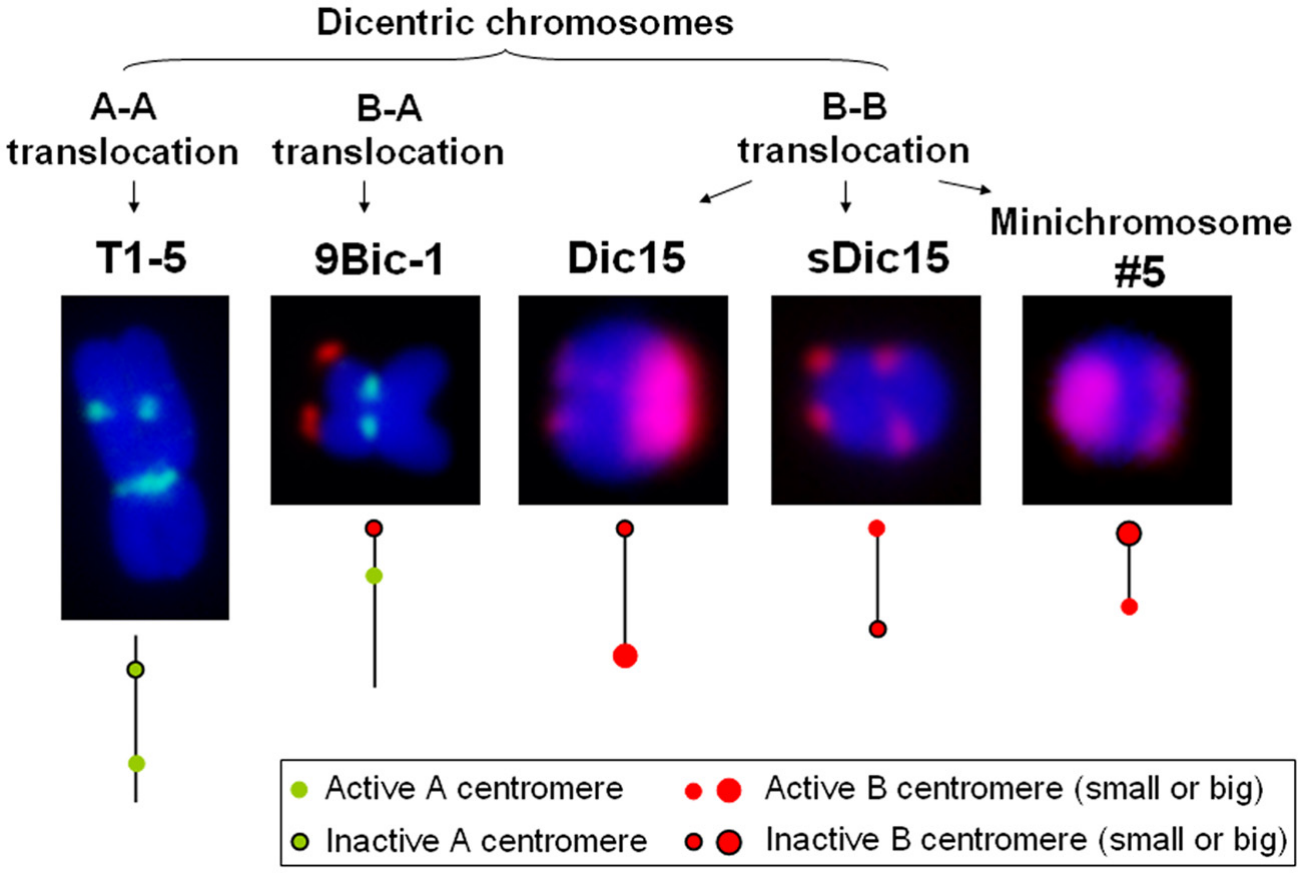
**Different kinds of dicentric chromosomes in maize.** Dicentric chromosome T1-5 is derived from an A-A translocation with an active centromere 1. 9Bic-1 is derived from a B-A translocation with active centromere 9. Dic15, sDic15, and minichromosome #5 are derived from combining B centromeres using a reverse duplication of 9S on the TB-9Sb translocation as described in the text. CRM is in green; B-repeat is red.

**FIGURE 2:**
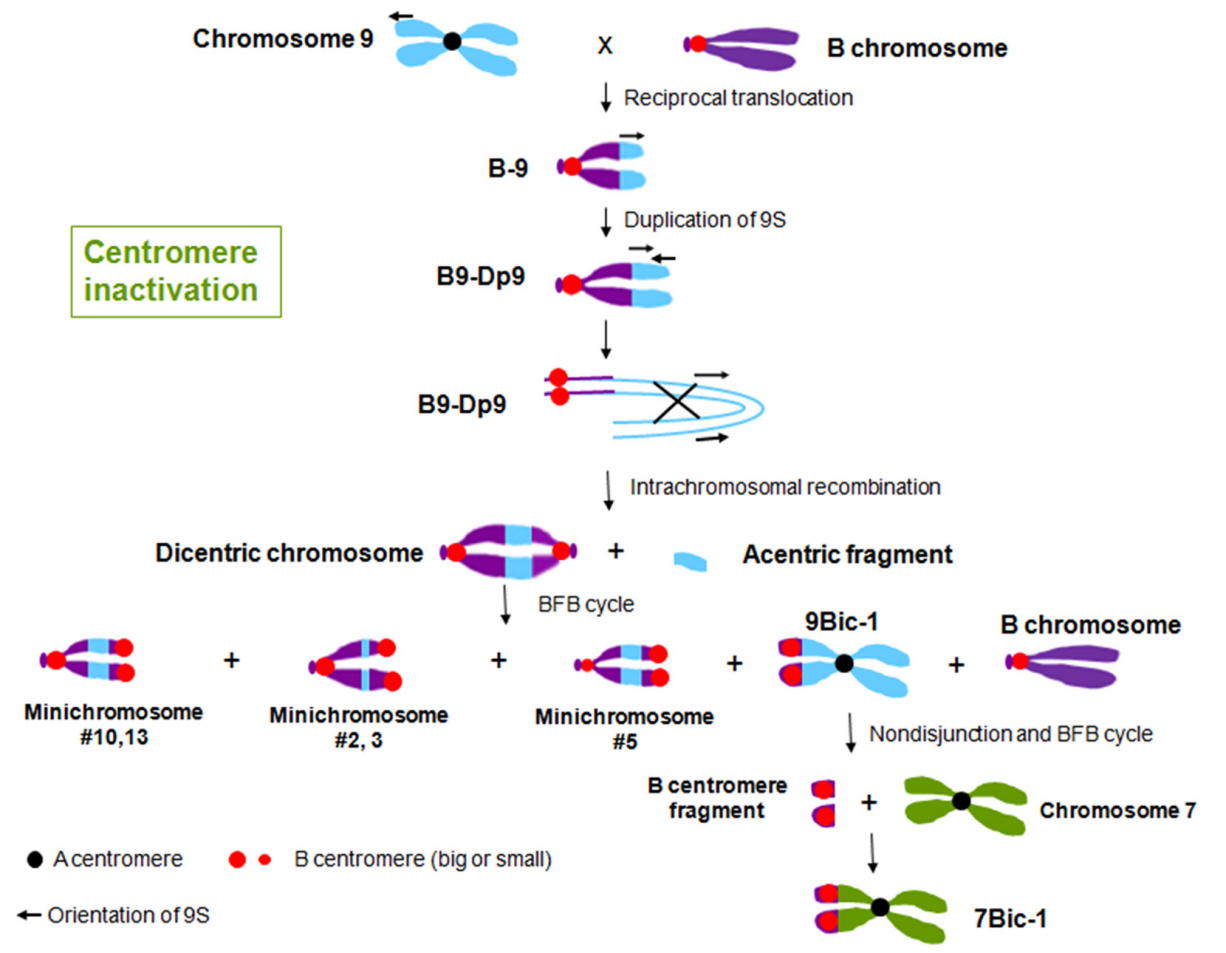
**Centromere inactivation through the process of BFB cycle in maize.** Reciprocal translocation between chromosome 9 and the B chromosome produced chromosome B-9, with an active B centromere and 9S. Duplication of 9S on B-9 generated chromosome B9-Dp9. The arrows indicate the orientation of the duplicated regions. Intrachromosomal recombination of B9-Dp9 produced dicentric chromosomes with two B centromere regions and a chromosomal fragment without a centromere region. This dicentric chromosome undergoes a BFB cycle, and produced different kinds of new dicentric chromosomes. Most of these dicentric chromosomes are with two B centromere regions, while in 9Bic-1, centromere 9 is active and the B centromere is inactive. The inactive B centromere region on 9Bic-1 remains adhered at the second pollen mitosis, which causes the chromosome to break. The broken piece formed a new A-B translocated chromosome 7Bic-1 with an inactive B centromere.

In maize, multiple dicentric chromosomes have been produced by chromosome translocation involving A and B centromeres, B and B centromeres as well as A and A centromeres (**Figure 1**). The B-A translocation chromosome B9-Dp9 was produced by the short arm of chromosome 9 (9S) being translocated to the B centromere, on to which the inverted duplication of 9S was recombined (Zheng et al., 1999; Kato et al., 2005). Recombination within B9-Dp9 can generate dicentric chromosomes with two identical B centromeres, and then this dicentric chromosome can undergo a Breakage-Fusion-Bridge (BFB) cycle. After several rounds of breakage and fusion, different kinds of newly formed dicentric mini-chromosomes are produced (Han et al., 2006; **Figure 2**). The configurations are distinct in these dicentric chromosomes: mini-chromosomes 2, 3, and 13 have two changed B centromere regions with similar sizes with one active; mini-chromosome 10 has multiple regions with centromeric DNA sequences while the functional centromere does not occupy all regions; and in mini-chromosome 5 the smaller centromere is functional rather than the larger one (**Figure 1**; Han et al., 2006). These mini chromosomes show patterns of centromere cohesion and disjunction during meiosis different from the normal ten pairs of A centromeres (Han et al., 2007a). The sister chromatids separate early at meiosis I in plants containing one copy of the mini chromosome, and the cohesion-mediating histone phosphorylation of H3S10 as well as the cohesion protector Shugoshin protein are still located on the separated mini chromosome in anaphase I (Han et al., 2007a). Among the dicentric derivatives of B9-Dp9, the B-A translocation chromosome 9Bic-1 was detected, with the B centromere transferred to the short arm of chromosome 9. The centromere 9 is active while the B centromere is inactive; this B-A translocation is an unexpected product from B-B dicentric formation that initiates a chromosome type BFB cycle (Han et al., 2006). In 9Bic-1, the process of non-disjunction of the B centromere in the presence of a whole B chromosome will cause chromosome breakage because the chromosome nine centromeres separate but the terminal B centromere sisters remain adhered to each other. This releases a fragment with the inactive B centromere. One such broken B centromere containing fragment attached to the short arm of chromosome 7 to generate a new dicentric chromosome 7Bic-1 (Han et al., 2007b; **Figure 2**). Apart from these dicentric chromosomes derived from the B centromere, an A-A translocation between chromosomes 1 and 5 in maize produced a structurally dicentric chromosome T1-5 (8041), which possesses an inactive centromere (Gao et al., 2011; **Figure 1**). In wheat, hybridization between wheat and rye generated a translocation line with two centromere regions (Fu et al., 2012). The same holds true for wheat-barley translocations, where one of the two centromeric regions on the translocated chromosomes became inactive (Nasuda et al., 2005).

**FIGURE 3:**
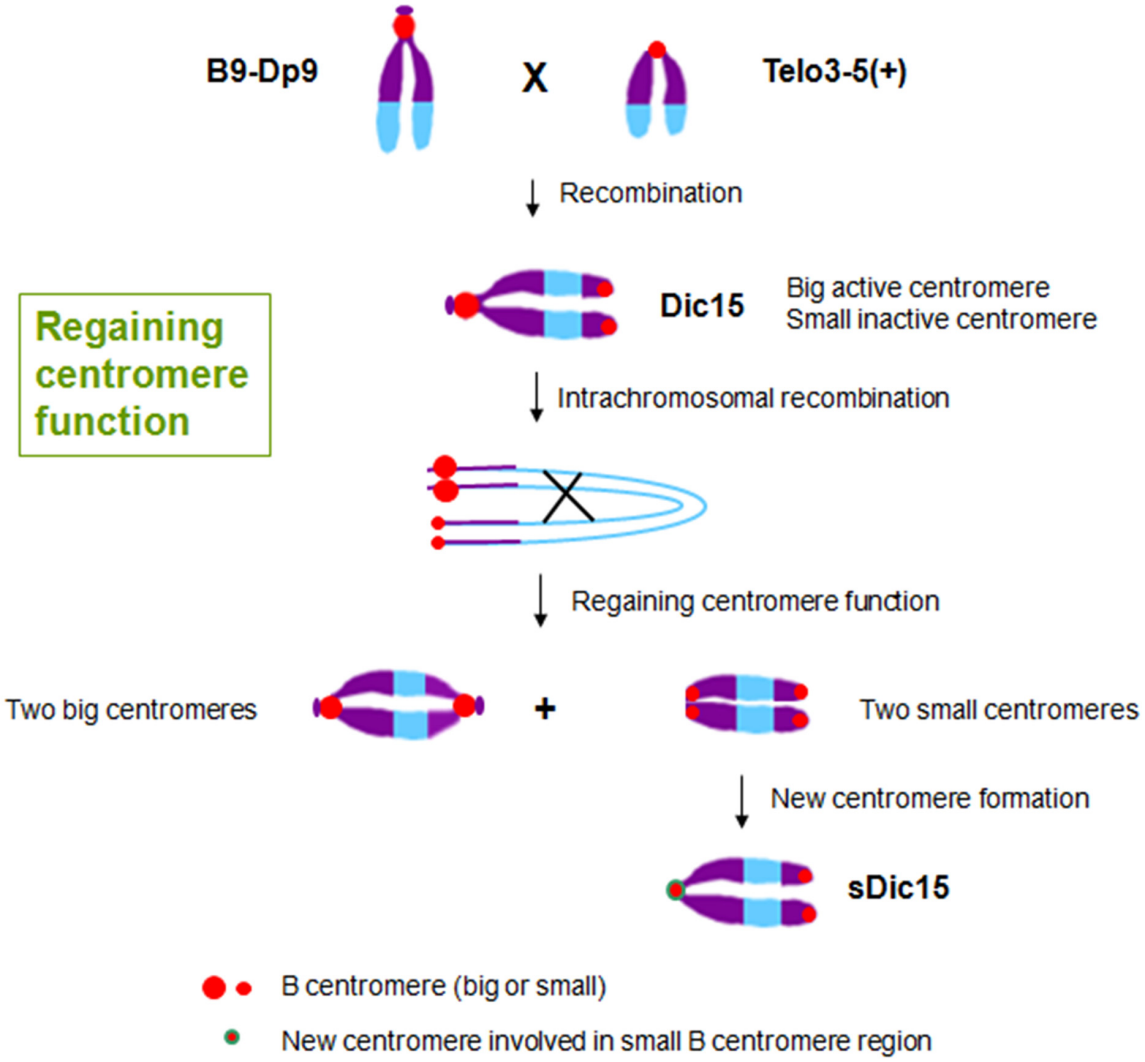
**Regaining centromere activity in maize.** The hybridization between plants with translocation chromosome B9-Dp9 and those with B centromere misdivision derivative T3-5(+) produced dicentric chromosome Dic15 with one big centromere and one small centromere with the small one inactive. Intrachromosomal recombination of Dic15 can generate new dicentric chromosomes with two big centromeres or two small centromeres. sDic15 is a dicentric chromosome with small centromeres that regained centromere activity via a 723 kb genomic DNA sequence *de novo* centromere.

The inactive centromeres have no function during cell division, and there is no CENH3 loading on the inactive centromeres (Han et al., 2006; Gao et al., 2011). As the original centromere sequences are unchanged in inactive centromeres, there are apparently epigenetic factors regulating CENH3-nucleosome loading only on active centromere regions (Birchler and Han, 2009). Epigenetic factors can also operate when centromere function is regained on a chromosome formerly with only inactive centromere regions. For example, when plants with the translocation chromosome B9-Dp9 (with a big B centromere region) were crossed with another carrying centromere misdivision derivative T3-5(+) (with a small B centromere region), dicentric chromosome Dic15 was produced and transmitted to the next generation with one big active centromere and one inactive small centromere (**Figure 3**). The joining of the two centromeres resulted in inactivation of the smaller one. Then intrachromosomal recombination of Dic15 produced new dicentric chromosomes with two small inactive centromeres or two large active centromeres. On the chromosome with two small centromeres, one of the originally inactive small centromeres appeared to be reactivated (Han et al., 2009; **Figure 3**). Among these new chromosomes with two sites of small centromere regions, sDic15 was found to have *de novo* centromere sequences as determined by CENH3 ChIP-seq. It is therefore possible that the regaining of centromere function on this chromosome is from a *de novo* centromere rather than reactivation of the B centromere sequence. There is no B centromere reference sequence to test whether the formerly inactive centromere sequences were involved in a reactivation process. The analysis showed that a 723 kb genome DNA sequence from the short arm of chromosome 9 was involved in the *de novo* centromere formation (Zhang et al., 2013a; **Figure 3**).

From study of dicentric chromosomes with one big centromere and one small centromere as determined by FISH for centromeric DNA amounts, it can be realized that centromere size does not determine centromere activity. In Dic15, the small centromere is inactive, while in mini-chromosome 5 derived from intrachromosomal recombination and BFB cycle of B9-Dp9, the small centromere is active (Han et al., 2006, 2009; **Figure 1**). For these two dicentric chromosomes, all centromeres have B-repeat containing regions. Minimal centromere size for function has been studied in maize using the system of centromere misdivison of the B centromere on chromosome B-9 (the chromosome containing the B centromere in TB-9Sb). It was revealed that chromosomes with estimated small centromeres have low transmission rate (Kaszás and Birchler, 1996, 1998). However, it is important to realize that the size of a centromere as determined by DNA amount might not necessarily reflect the centromere chromatin domain size. At present, it is not possible to make generalization about centromere inactivation, which potentially could be stochastic.

In an active centromere, the chromatin maintains an open state for CENH3-nucleosome deposition dynamically in the cell cycle, but in the inactive centromere, the chromatin state is closed. The arrangement order and higher structure of centromeric DNA sequence may provide the basic structure for centromere establishment, while epigenetic elements including histone modifications and chromatin assembly factors are the determinants for CENH3-nucleosome loading (Ekwall, 2007; Allshire and Karpen, 2008; Black and Cleveland, 2011). These epigenetic elements may create on or off states of centromeric chromatin, so that CENH3-nucleosomes can load or fail to load onto the centromere region (Svensson and Ekwall, 2014). In yeast and human, several factors involved in centromere assembly have been reported, including histone deacetylation (Sato et al., 2012), centromere histone H2B monoubiquitination (Sadeghi et al., 2014), CENP-A Ser68 phosphorylation (Yu et al., 2015), and K124 ubiquitylation (Niikura et al., 2015). However, in maize, such factors have not been identified.

## Centromere Non-Disjunction

The centromere of the maize B chromosome can undergo non-disjunction at the second pollen mitosis, and the sperm with B chromosomes will preferentially fertilize the egg in the process of double fertilization (Roman, 1947, 1948; Carlson, 2007). There are factors on the B chromosome that are required for non-disjunction of the B centromere, apparently including the B centromere adjacent heterochromatin, a site in proximal euchromatin and another at the very distal tip of the long arm (Roman, 1947; Lin, 1978). In the B-A translocation chromosomes 9Bic-1 and 7Bic-1, the inactive B centromere is transferred to the short arm of chromosome 9 or chromosome 7, respectively. These inactive B centromeres can still perform non-disjunction in the presence of a whole B chromosome; thus non-disjunction does not rely on centromere function. Furthermore, the knob heterochromatin region near the B centromere is deleted in 9Bic-1 and 7Bic-1, so it is likely that the B specific centromere sequence is responsible for non-disjunction given that it is the only major unique repeat unit remaining on these chromosomes (Han et al., 2007b). The inactive B centromere state does not affect non-disjunction, indicating the sequence plays a role in non-disjunction. However in rye, the non-disjunction of the B chromosome relies on B centromere function; the pericentromere cohesion is related to B centromere non-disjunction (Banaei-Moghaddam et al., 2012).

## Centromere Pairing

**FIGURE 4:**
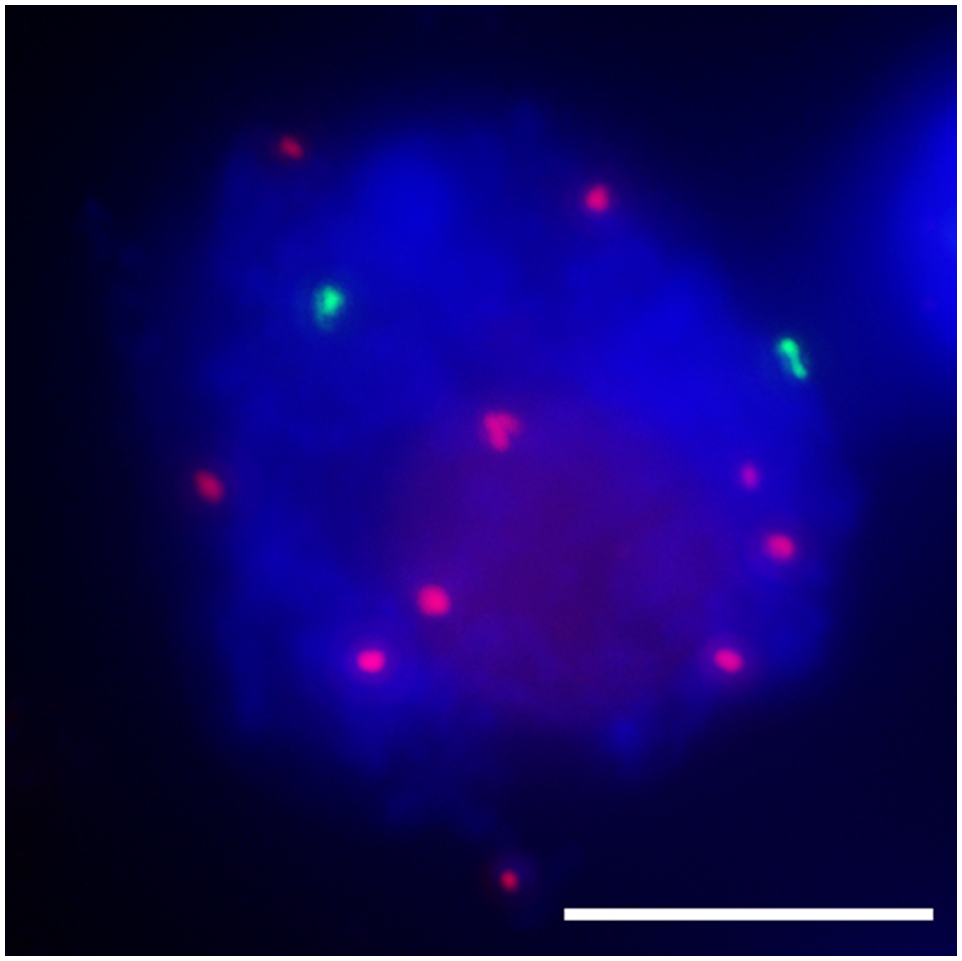
**Inactive centromeres (7Bic-1 in green) can not pair at the leptotene stage, when the ten pairs of functional centromeres pair completely.** Immuno-FISH using an antibody against centromeric histone CENH3 in red and B repeat probe in green; chromosomes are counterstained with DAPI in blue. The inactive B centromeres are separated but the ten active A centromeres are all paired. Bar = 10 μm.

**FIGURE 5:**
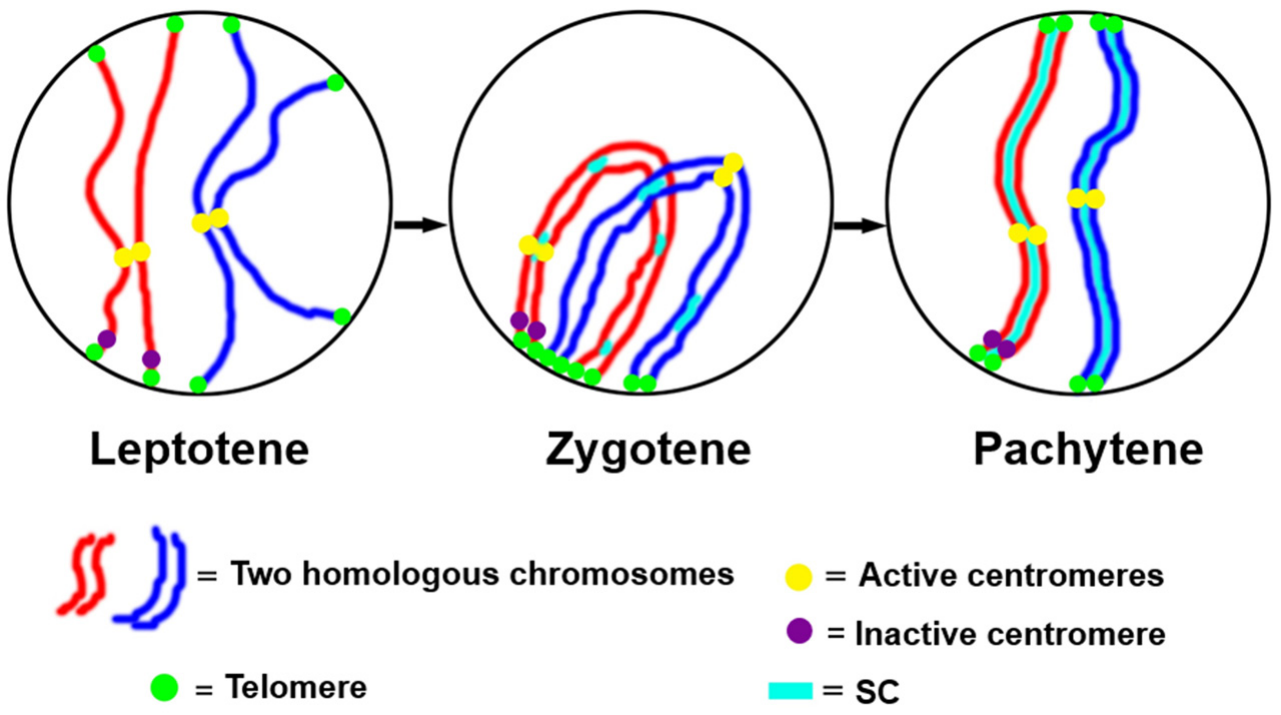
**A model for centromere pairing in meiotic early prophase I in maize.** We suggest a model of homologous chromosome initiation in maize in which homologous chromosomes find each other at the leptotene stage and are recognized at centromeres via a centromere function dependent mechanism. The inactive centromeres do not participate in this pairing. Then chromosome pairing is further facilitated by telomere bouquet formation and synaptonemal complex (SC)-dependent pathways.

The function of the centromere in chromosome orientation and segregation during the cell cycle has been studied in detail. In maize, homologous chromosome pairing is initiated from centromeres, and centromere pairing occurs prior to telomere bouquet formation (Zhang et al., 2013b). It was revealed that function rather than DNA sequence is responsible of centromere pairing in maize, suggesting that dynamic chromosome binding factors at active centromeres may take part in the homologous pairing process. In 7Bic-1, the inactive B centromere regions are not paired, when other active centromeres are paired completely at the leptotene stage (**Figure 4**). Compared to the chromosome arm regions and pericentromere sites, functional centromere regions are the earliest recognition points for homologous chromosomes. The synaptonemal complex (SC) and sister-chromatid cohesion are required for centromere pairing, suggesting that they are involved in homologous centromere recognition (Zhang et al., 2013b; **Figure 5**).

## *De Novo* Centromere Formation

Inactive centromeres show that DNA sequence alone can not determine centromere establishment. Furthermore, a specific DNA sequence is not always required for centromere assembly. Additional evidence for epigenetic effects on centromere specification comes from the recognition that *de novo* centromeres can be formed on acentric chromosomal fragments without canonical centromeric repeat sequences produced by chromosome breakage. It has been proposed that a tug of war between typically larger endogenous centromeres and smaller *de novo* ones inactivate the latter (Liu et al., 2015), clearing it from the arm in analogous fashion to the generation of Dic15 described above when a small centromere was placed on a dicentric with a normal sized endogenous centromere and became inactive (Han et al., 2009).

**FIGURE 6:**
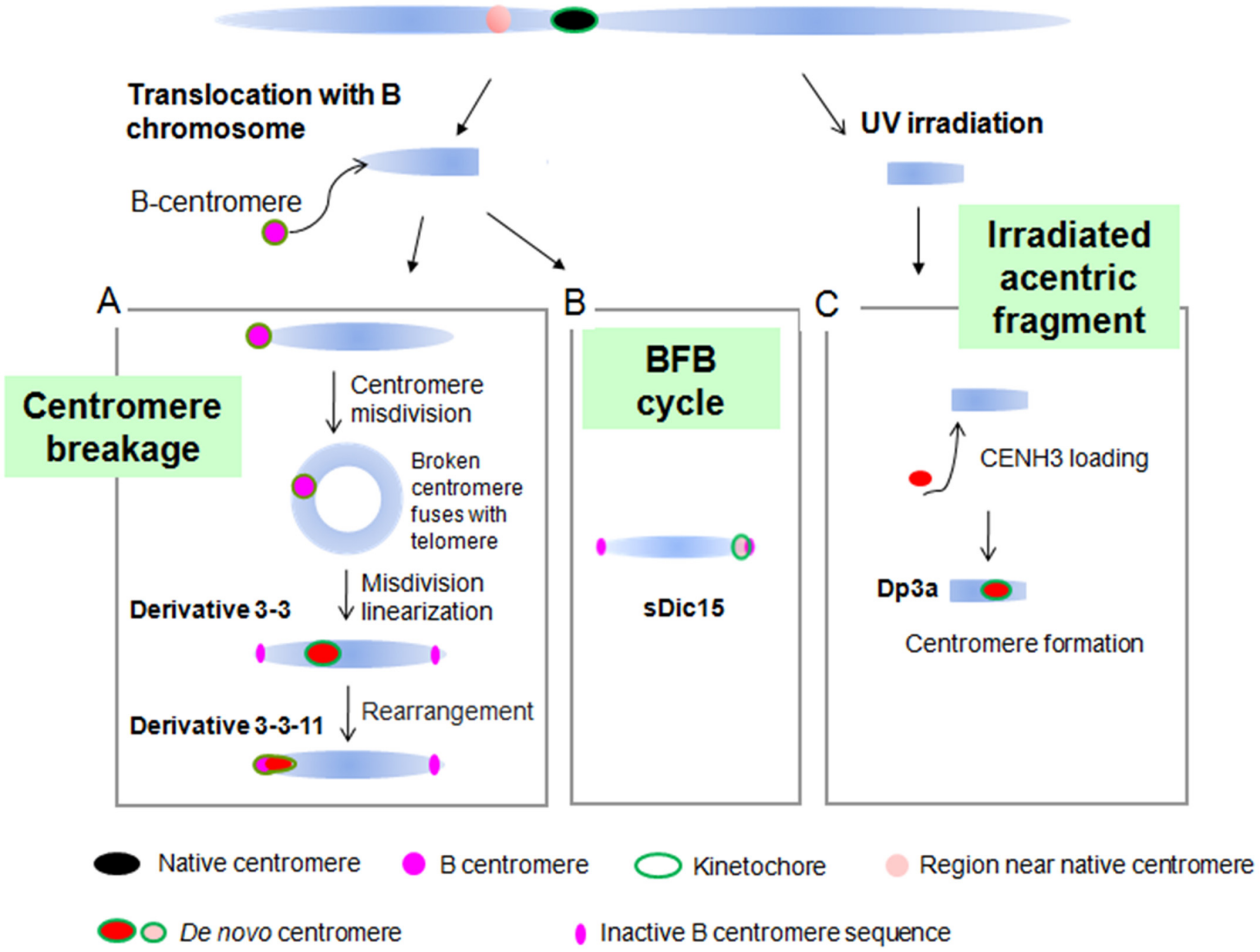
***De novo* centromere formation in maize.** (A) *De novo* centromere generated by centromere misdivision. B centromere misdivision on the B-9 chromosome causes a broken centromere that fuses with the telomere region of the same chromosome to produce a ring chromosome. The ring chromosome broke and became linear and gained a 288 kb *de novo* centromere in derivative 3-3. In the derivative of 3-3, this *de novo* centromere was inactivated and another 200 kb *de novo* centromere arose. (B) sDic15 has a *de novo* centromere derived from a 723 kb sequence. (C) Irradiated acentric chromosomal fragments can acquire a *de novo* centromere by CENH3 seeding at that position. Chromosome fragment Dp3a has a 350 kb *de novo* centromere from a sequence on the long arm of chromosome 3.

*De novo* centromeres have been found in several plant species. In a wheat background, a stable 7HS telosome with no detectable barley or wheat centromeric sequences was generated (Nasuda et al., 2005). In maize, several *de novo* centromeres have been found. On the chromosomal fragment Duplication 3a (Dp3a), a *de novo* centromere appeared with a 350 kb CENH3 binding region from the long arm of chromosome 3 (Fu et al., 2013; **Figure 6**). The dicentric chromosome sDic15, mentioned above, contains 723 kb of genomic DNA sequence from the short arm of chromosome 9 in the active centromere, with a similar DNA methylation level and DNA composition to the native centromere regions (Zhang et al., 2013a; **Figure 6**). By the process of the B centromere misdivision in maize translocation line TB-9Sb (Kaszás and Birchler, 1998), chromosome derivative 3-3 was produced with a 288 kb *de novo* centromere derived from the distal region of the short arm of chromosome 9. In subsequent derivatives of 3-3, the *de novo* centromere of 3-3 was inactivated with another *de novo* centromere formed in 3-3-11 with 200 kb sequence on the short arm of chromosome 9. These *de novo* centromeres are derived from different regions and occupy different sequences (Liu et al., 2015; **Figure 6**). Two isolates of maize chromosome 3 contained neocentromeres near the original centromere 3 when normal maize chromosomes were transfered to an oat background (Wang et al., 2014).

The above-mentioned cases indicate that *de novo* centromere formation is common in maize. The large native centromere appears capable of suppressing potential *de novo* centromeres forming on chromosome arms. Potential *de novo* centromeres be established only on fragments without native centromeres. Any process that produces chromosomal fragments can potentially promote *de novo* centromere formation on the otherwise acentric chromosome fragments.

The sites for neocentromere formation are not related and the mechanism of centromere formation at ectopic regions is still a mystery (Scott and Sullivan, 2014). In maize, *de novo* centromeres derived from the sequences near active centromere regions or on the distal regions of chromosomal arms have been found (Fu et al., 2013; Zhang et al., 2013a). The sizes of *de novo* centromeres vary as noted above, but the smallest, which is only 200 kb, can transmit stably in meiosis (Liu et al., 2015). Maize centromeres sizes were dramatically expanded and adopt a uniform size in the genetic background of oat, which have larger centromere chromatin domains than maize (Wang et al., 2014).

For many *de novo* centromeres in various species, no common DNA features or DNA motifs for centromere deposition have been found (Birchler et al., 2011). In sDic15, there are two DNA motifs enriched in the *de novo* centromere that are also in native centromeres (Zhang et al., 2013a), but in other *de novo* centromeres, such motifs are not recognized. After the native centromere is deleted on a chromosomal fragment, which kinds of factors are involved in choosing a specific region for CENH3 seeding and centromere establishment are still not known. Most acentric fragments are lost during cell division because there is no CENH3 loading.

## DNA Methylation in Centromeric Region

DNA methylation level is important for centromere identity and function through regulating the centromeric chromatin state (Choo, 2001; Schueler and Sullivan, 2006). The DNA methylation level has been determined in *de novo*, active and inactive centromeres in maize. The CENH3-nucleosome binding DNA sequences in native centromeres of maize is hypomethylated compared to the DNA sequences associated with the flanking pericentromere, as is the case in *Arabidopsis thaliana* (Zhang et al., 2008). For the B chromosome centromere, hypomethylated DNA also exists in the active B centromere, and the inactive B centromere has hypermethylated DNA (Koo et al., 2011). Core centromere chromatin is not heterochromatin; to some degree it has a loose structure for CENH3-nucleosome deposition. The hypomethylated DNA in centromere regions may provide a relaxed chromatin environment to allow centromeric transcription and also serve as a marker recognized by other factors for centromere assembly.

As centromere formation and maintenance of centromeric chromatin are regulated by epigenetic factors, the methylation modification on centromere DNA may take part in centromere chromatin assembly (Gopalakrishnan et al., 2009). The DNA methylation in centromere regions may influence the transcription state, and transcription in a centromere may play a role in CENH3-nucleosome assembly (Allshire and Karpen, 2008).

## Transcription in Centromere Regions

Centromere transcripts from the repeat sequences have been found in many species. These transcripts are essential for regulating CENH3 nucleosomes loading in the centromere region (Chan and Wong, 2012) and promoting kinetochore complex assembly (Scott, 2013). In maize, transcripts from centromere repeat sequences CentC and CRM2 have been detected (Topp et al., 2004; Du et al., 2010) and the CentC RNA can interact with CENP-C (Du et al., 2010). In the *de novo* centromere sDic15, several genes inside the 723 kb centromere region are transcribed (Zhang et al., 2013a). In a neocentromere of human, the transcript of retrotransposon LINE 1 was required for stable transmission of the neocentromere during the cell cycle (Chueh et al., 2009). As *de novo* centromeres have no traditional centromere repeat sequences, the transcripts in *de novo* centromeres should be different from the native centromeres.

The function of centromere transcription in CENH3-nucleosome assembly has been studied. In *Drosophila melanogaster*, non-coding RNA of satellite III in the centromere region is required for deposition of CENP-A nucleosomes and CENP-C (Rosic et al., 2014). The work using an ectopic centromere system in *Drosophila* showed that centromere transcription is required for CENP-A deposition, and the production of these transcripts is dependent on CENP-A chaperone recruiting chromosome assembly factor and RNA polymerase II to the centromere region (Chen et al., 2015). The transcripts of centromere satellite in human interacts with CENP-A and its chaperone Holliday junction recognition protein (HJURP; (Quenet and Dalal, 2014).

The function of centromere transcripts in CENH3-nucleosome loading can be divided into two parts. Firstly, the chromosome assembly factor as well as histone chaperone and RNA polymerase II are recruited to the centromere region to produce an open chromatin state for CENH3-nucleosome loading. Then the transcribed RNA can interact with CENH3 and the chaperone as well as kinetochore proteins to promote CENH3 assembly and maintain the centromeric chromatin. In dicentric chromosomes, the transcripts from an active centromere cannot work in the inactive centromere region, and may result from the relatively closed chromatin state in the inactive centromere.

## Summary

DNA sequence and histone modification are involved in formation and maintenance of centromere activity. Centromere DNA is the carrier for centromere chromatin. *De novo* centromere formation is common in maize. Centromere inactivation shows that apart from histone variants and histone modifications, other elements including histone chaperones and chromatin assembly factors as well as transcription factors are working in regulation of centromere chromatin. The transcription products may be combined in the centromere region controlling CENH3-nucleosome loading.

Centromere DNA can play roles independent of centromere function in non-disjunction of the B centromere in maize, in that specific products emanating from a whole B chromosome can act in trans on the inactive B centromere to induce non-disjunction (Han et al., 2007b). When the higher structure of centromeric chromatin is studied in more depth, the nature of non-repeat DNA sequence in *de novo* centromeres and the highly repetitive DNA sequences in native centromeres as well as how the centromere DNA sequence is involved in centromere formation and centromere non-disjunction will be understood better.

An active centromere is required for homologous chromosome pairing in maize (Zhang et al., 2013b). The key factors controlling homologous chromosome pairing may locate in centromere regions in early meiosis. Thus, the function of the centromere is reflected not only in chromosome orientation and segregation, but also in chromosome recognition and genome stability.

## Key Concepts of Centromere Function in Maize

### Concept One: Centromere

The centromere is the major constriction on a metaphase chromosome. In most species, it is composed of repeat sequences. Accurate assembly of the kinetochore complex is dependent on functional centromeric chromatin, and the correct spindle attachment on the centromere region ensures accurate chromosome orientation and segregation.

### Concept Two: Dicentric Chromosomes

The chromosomes with two sites of centromere sequences are called dicentric chromosomes. In order to remain stable, one centromere must be active while the other centromere must become inactive. Otherwise chromosomes with two functional centromeres would be unstable during cell division if the two centromeres separate in opposite directions.

### Concept Three: Centromere Inactivation

The process of centromere state change from active to inactive is called centromere inactivation. The inactive centromere loses CENH3, while the centromeric DNA sequence is still present. There is no spindle contact to the inactive centromere during cell division, and the dicentric chromosome can transmit stably.

### Concept Four: Centromere Non-disjunction

At the second pollen mitosis, the maize B sister centromeres are held together so that they are transferred to the same pole at anaphase. Such non-disjoined chromatids preferentially transmit to the egg cell. In the presence of the whole B chromosome, an inactive B centromere region can perform non-disjunction.

### Concept Five: Centromere Pairing

In maize, during early meiotic prophase, centromere associations occur between homologous chromosomes before telomere bouquet formation and chromosome arm pairing. Homologous chromosomes recognize each other through centromere regions. An active centromere is necessary to participate in this process.

### Concept Six: *De Novo* Centromere

*De novo* centromeres are newly formed centromeres in otherwise non-centromeric regions on chromosomes without traditional centromeric repeat sequences. *De novo* centromeres form on chromosomal fragments generated through different processes, such as chromosome rearrangements from centromere misdivision, BFB cycle and so on, in which the native centromere has been deleted or inactivated.

## Conflict of Interest Statement

The authors declare that the research was conducted in the absence of any commercial or financial relationships that could be construed as a potential conflict of interest.
